# LINC01117 inhibits invasion and migration of lung adenocarcinoma through influencing EMT process

**DOI:** 10.1371/journal.pone.0287926

**Published:** 2023-06-29

**Authors:** Linjun Liu, Wenjia Ren, Licheng Du, Ke Xu, Yubai Zhou

**Affiliations:** 1 Department of Environment and Life Sciences, Beijing University of Technology, Beijing, China; 2 NHC Key Laboratory of Biosafety, National Institute for Viral Disease Control and Prevention, Beijing, China; Columbia University Irving Medical Center, UNITED STATES

## Abstract

**Background:**

Studying the mechanism of action of LncRNAs in lung adenocarcinoma (LUAD) is of great importance for an in-depth understanding of the molecular mechanism of lung adeno carcinogenesis and development.

**Objective:**

The aim is to identify a long non-coding RNA LINC01117 that is specifically and highly expressed in LUAD cells and to investigate its biological functions and molecular mechanisms in LUAD cells, providing a new potential target for targeting LUAD therapy.

**Methods:**

This study used publicly available data downloaded from The Cancer Genome Atlas (TCGA) database. Construction of siRNA and overexpression plasmid-packed lentiviral constructs were used to knock down and increase the expression of LINC01117 in LUAD cells. The effect of LINC01117 on LUAD cell migration and invasion was verified by scratch assays and Transwell assays. Western blot assays were performed to verify the effect of knocking down LINC01117 expression on key proteins of the EMT process. The effect of overexpression and knockdown LINC01117 expression on key proteins of the EMT process and the nuclear and cytoplasmic distribution of YAP1, a key effector molecule of the Hippo pathway, was verified by Western blot assays.

**Results:**

LINC01117 expression was upregulated in LUAD tissues and cell lines. Clinical correlation and prognostic analyses showed that LINC01117 was associated with poorer clinical features (staging and N classification) and poorer prognosis and could be analyzed as an independent prognostic factor. Cell migration and invasion were significantly inhibited in the knockdown group compared to the control group; in contrast, cell migration and invasion were promoted in the overexpression group. Overexpression of LINC01117 resulted in down-regulation of E-cadherin expression and increased expression levels of N-cadherin, vimentin, ZEB1, snail and slug; in contrast, knockdown of LINC01117 appeared to have the opposite effect. Furthermore, knockdown of LINC01117 increased the enrichment of YAP1 protein in the cytoplasm and reduced its level in the nucleus; overexpression of LINC01117 produced the opposite intracellular distribution results.

**Conclusions:**

LINC01117 was highly expressed in LUAD, and knockdown of LINC01117 significantly inhibited the migration and invasion of LUAD cells, while overexpression of LINC01117 significantly promoted the migration and invasion of LUAD cells, and affected the EMT process, and was able to alter the distribution of YAP1 in the nucleus and cytoplasm. This suggests that LINC01117 may regulate the activity of the Hippo pathway by altering the nuclear and cytoplasmic distribution of YAP1, which in turn induces the EMT process in lung adenocarcinoma cells and thus exerts a pro-cancer effect. It suggests that LINC01117 may play a key role in the occurrence and development of LUAD.

## Introduction

Lung cancer is a malignant tumors with extremely high morbidity and mortality rates worldwide, seriously affecting human health [1]. According to statistics, there will be approximately 2.2 million new cases and 1.8 million deaths of lung cancer worldwide in 2020, the highest cancer incidence and mortality rate among men and the second highest cancer incidence rate among women, second only to breast cancer [2]. Based on pathology, they can be divided into two main categories: small cell lung cancer and non-small cell carcinoma lung cancer, which can be subdivided into adenocarcinoma of the lung, squamous lung cancer and large cell carcinoma [3]. LUAD is the most prominent tissue subtype of non-small cell lung cancer (NSCLC), with high mortality and metastasis rates [4]. LUAD accounts for 40% of all lung cancers [5] and is commonly found in non-smokers and young women [6], mainly originating from the bronchial mucosal epithelium and bronchial mucus glands, and can occur in the small bronchi or central airways, mostly presenting as peripheral lung cancer, prone to local infiltration and early metastasis [7]. Immunotherapy is often used in advanced stages. Although there have been some improvements in the techniques and tools used to treat LUAD [8, 9], the five-year survival rate for patients is still less than 4% [10, 11].

Most of the RNAs in the human genome cannot be translated into proteins, and these are called non-coding RNAs [12]. Some non-coding RNAs are greater than 200 nucleotides in length and are called long non-coding RNAs (LncRNAs). Among them, long intergenic non-coding RNAs (LncRNAs) are a class of long-stranded non-coding RNAs that lie between coding genes and are usually smaller than protein-coding transcripts [13]. LncRNAs are expressed in low amounts but exhibit strong tissue specificity. Studies have shown that LncRNAs play important regulatory roles in epigenetic, cell cycle and differentiation and other life activities [14, 15].

In this study, a long-stranded intergenic non-coding RNA LINC01117, located on chromosome 2 and 656bp in length, was identified by bioinformatics analysis in the previous phase. Wang et al. found that LINC01117 could be developed as a biomarker for breast cancer patients through biomarker analysis [16], however, the biological function of LINC01117 in tumors and its molecular mechanism of action have not yet been reported. We found that LINC01117 expression was significantly upregulated in LUAD patient tissues and that high LINC01117 expression was negatively correlated with the prognosis of LUAD patients. We examined the effect of LINC01117 expression on the migration and invasive ability of LUAD cells by knocking down and overexpressing LINC01117, and we investigated the molecular mechanism of LINC01117 in the development of LUAD. In conclusion, LINC01117 as an oncogene promotes the development of lung cancer and through our study LINC01117 is expected to be a biomarker for lung cancer diagnosis and a potential therapeutic target.

## Materials and methods

### Materials

Human non-small cell carcinoma lung cancer cell lines (H1650, H1299, A549, H460) and immortalized normal human bronchial epithelial cells 16HBE are kept in our laboratory. Dulbecco’s Phosphate Buffer (DPBS), RPMI-1640, Dulbecco’s Modified Eagle Media (DMEM), fetal bovine serum (FBS), 100×Pen Strep, 2.5% trypsin-EDTA were purchased from Gibco, USA. The reverse transcription and qRT-PCR kits were purchased from Takara; Trizol was purchased from ambion; trichloromethane, anhydrous ethanol and isopropanol were purchased from Beijing Chemical Factory; PMSF was purchased from Beijing Solarbio Technology Co. protein loading buffer, 20× protein transfer buffer, 10× SDS-PAGE electrophoresis buffer, and blocking solution were purchased from Beyotime Biotechnology Co. GAPDH, E-cadherin, N-cadherin, and vimentin antibodies were purchased from Cell Signaling Technology (Danvers, MA, USA); RNA-free enzyme water, endotoxin-free plasmid macro extraction kit, and plasmid micro extraction kit were purchased from Beijing TIANGEN Biochemical Co.

### Methods

#### Open access data acquisition and analysis

A dataset including RNA sequencing data from 535 LUAD tumors and 59 non-tumors tissues, as well as other relevant clinical information, was downloaded from The Cancer Genome Atlas website (TCGA, https://gdc-portal.nci.nih.gov/). Differential expression analysis of RNA sequencing data was performed using the Wilcox test with R software. The clinical indicators of the samples in the LUAD cohort, such as gender, age, T, N, M and tumor stage, were classified into two groups. Wilcox test was used to analyze whether the expression level of the above prognostic genes was different between the two groups of different clinical indicators. These were analyzed as independent prognostic factors for LUAD by univariate and multifactorial Cox regression.

#### Cell culture

H1650, H1299 and 16HBE were maintained in 1640 medium and A549 and H460 cells were maintained in DMEM medium, all media were supplemented with 10% fetal bovine serum and penicillin 100 U/mL and streptomycin 100 mg/mL and incubated in a humidified incubator at 37°C and 5% CO2.

#### Cell transfection

LINC01117-siRNA sequence was synthesized by Beijing tsingke Biotechnology. Cells were inoculated into six-well plates the day before transfection and replaced with 2ml of antibiotic-free medium containing 10% FBS on the following day; the transfection system was prepared by adding LINC01117-siRNA to 200ul of transfection buffer to a final concentration of 50mMol/mL, shaking and centrifuging, followed by shaking and centrifuging with 4uL of transfection reagent and incubating for 10min at room temperature. And all of them were added to the cells in the six-well plate, and the control and experimental groups were set up. The transfected cells were incubated in a humidified incubator at 37°C and 5% CO2 for 24h-72h.

#### Lentiviral packaging and infection

The packaging plasmid (psPAX2), envelope plasmid (pMD2.G), and shuttle plasmid containing LINC01117 target gene were synthesized by Beijing tsingke Biotechnology Company. HEK293T cells were inoculated in 100 mm cell culture dishes with 3×10^6^ cells per dish. 24 h later, when the cells reached 80% confluence, plasmid transfection was performed and plasmids were added according to the instructions (shuttle plasmid: psPAX2: pMD2.G = 4:3:1). After 48 h of transfection, the supernatant was collected and 10 mL of DMEM complete medium was replenished in a Petri dish. The supernatant was centrifuged at 3000 rpm, filtered through a 0.45μm membrane and stored at 4°C. After 24 h, the supernatant was collected again, centrifuged at 3000 rpm and filtered through a 0.45μm membrane. The supernatant was mixed and centrifuged at 100 000 g for 2 h at 4°C. The supernatant was carefully removed and the virus precipitate was suspended using 500μL DMEM and stored frozen at -80°C in a freezer.

#### Total RNA extraction and reverse transcription

The cells were washed with DPBS and the supernatant was aspirated; 500ul of trizol reagent was added to each well, repeatedly blown for 10min at room temperature, Trizol lysate and the lysed cells were transferred together into an ep-tube without RNase, one-fifth of Trizol chloroform was added, i.e. 100uL. Mix well by shaking vigorously for 15s, leave at room temperature for 10 min, centrifuge at 12000×g, 4°C for 15min; remove the upper aqueous phase into a new ep tube, add an equal volume of isopropanol and mix well, leave at room temperature for 10 min, centrifuge at 12000×g, 4°C for 10 min; discard the supernatant, add an equal volume of 75% ethanol as Trizol, wash with gentle blowing and centrifuge at 12000×g, 4°C for 5 min. Centrifuge for 5min; discard supernatant and allow to dry at room temperature; add RNase Free H2O to dissolve the precipitate when it changes from milky white to translucent, mix well and measure the concentration using an ultraviolet photometer. The cDNA was then reverse transcribed and stored at -20°C in the refrigerator.

#### Real time PCR

RNA levels were quantified using SYBR Green PCR Master Mix, with GAPDH as the internal reference gene, and the 2^-ΔΔCT^ rule was used to calculate the relative expression of both. qRT-PCR primer sequences were as follows: GAPDH: Forward GTCTCCTCTGACTTCAACAGCG, Reverse Sequence ACCACCCTGTTGCTGTAGCCAA; LINC01117 Forward: GAAGUCACUGAGACACCAATT, LINC01117 Reverse: UUGGUGUCUCUCAGUGACUUCTT.

#### Wound healing assay

Cells were inoculated in six-well plates at approximately 4×105 cells per well; the next day the plates were scored perpendicular to the tip of a 10μL gun. The cells were washed 3 times with PBS to remove the scratched cells, medium containing 1% FBS was added and the width of the scratch was recorded under the microscope for 0h and recorded as W0h. The width of the scratch was recorded as W24h after incubation in a humidified incubator at 37°C 5% CO2 for 24h. Mobility was calculated using Image J and plotted using the formula: Mobility = (W0h - W24h)/W0h.

#### Transwell assay

Cells were suspended in serum-free medium and counted, and prepared into 2.5–5×105/ml cell suspension; inoculation of cells: Transwell chambers were placed in 24-well plates, and 200μL of cell suspension was added to the upper chamber (for invasion experiments, matrix gel was spread in the chambers in advance, and the matrix gel was mixed with serum-free medium at a ratio of 1:9 and dropped into the Transwell, the cells were incubated in a 37°C cell incubator for more than 2h, so that the matrix gel was denatured and solidified by heat). 500μL of complete medium containing 10% FBS was added to the lower chamber of the 24-well plate and incubated in a humidified constant temperature incubator at 37°C with 5% CO2 for 24–48 h. The cells were fixed with 4% paraformaldehyde for 15 min and stained with crystal violet for 15 min. The cells and crystalline violet were removed from the upper chamber, dried for a few minutes and observed under the microscope. 3 randomly selected fields of view were photographed and all the cells in the photographed fields of view were counted, and the number of Transwell in each group of cells was calculated to characterize the migration and invasion ability of the cells.

#### Western blot

Cytoplasmic/nuclear protein extraction: Cells grown in logarithmic phase were collected by centrifugation, leaving the cell precipitate to be prepared. Add PMSF-added Cytoplasmic Protein Extraction Reagent A. Vortex vigorously for 5s at maximum speed to completely suspend and disperse the cell precipitate. Add Cytoplasmic Protein Extraction Reagent B. Vortex at maximal speed for 5s, ice bath for 1min. Vortex at maximal speed for 5s. Centrifuge and immediately aspirate the supernatant, which is the cytoplasmic protein extracted. For the precipitate, the residual supernatant is completely aspirated and the PMSF-added nucleoprotein extraction reagent is added. The supernatant is immediately aspirated by centrifugation and the nucleoprotein is extracted. Total protein extraction: Cells were lysed on ice with cell lysis solution spiked with protease inhibitor, and cell debris and supernatant were separated by centrifugation at 12,000r/min for 5min at 4°C. The supernatant was taken and the total protein concentration was determined using the BCA method. The protein samples were added to the appropriate amount of loading buffer, boiled in a water bath for 5min and separated by 7.5%-12% SDS-PAGE, and the membranes were transferred at constant flow for 2h. The PVDF membranes with transferred proteins were closed in fast closing solution for 30min, incubated overnight at 4°C with proportionally diluted primary antibody, washed three times in TBST and incubated with horseradish peroxidase-associated secondary antibody (1:1000) After washing with TBST, the ECL chemiluminescent solution was reacted with a gel imager, and the results of the protein bands were photographed and the grey scale values were calculated using ImageJ.

#### Statistical analysis

All data were expressed as mean ± standard deviation (x±s), and the experimental data were statistically processed using the software GraphPad Prism 8.0. Comparisons between multiple groups were made using ANOVA, and comparisons between two groups were processed using t-test. p<0.05 indicates a significant difference with statistical significance.

## Results

### LINC01117 expression was upregulated in LUAD tissues and correlated with aggressive clinical features

**Fig 1:**
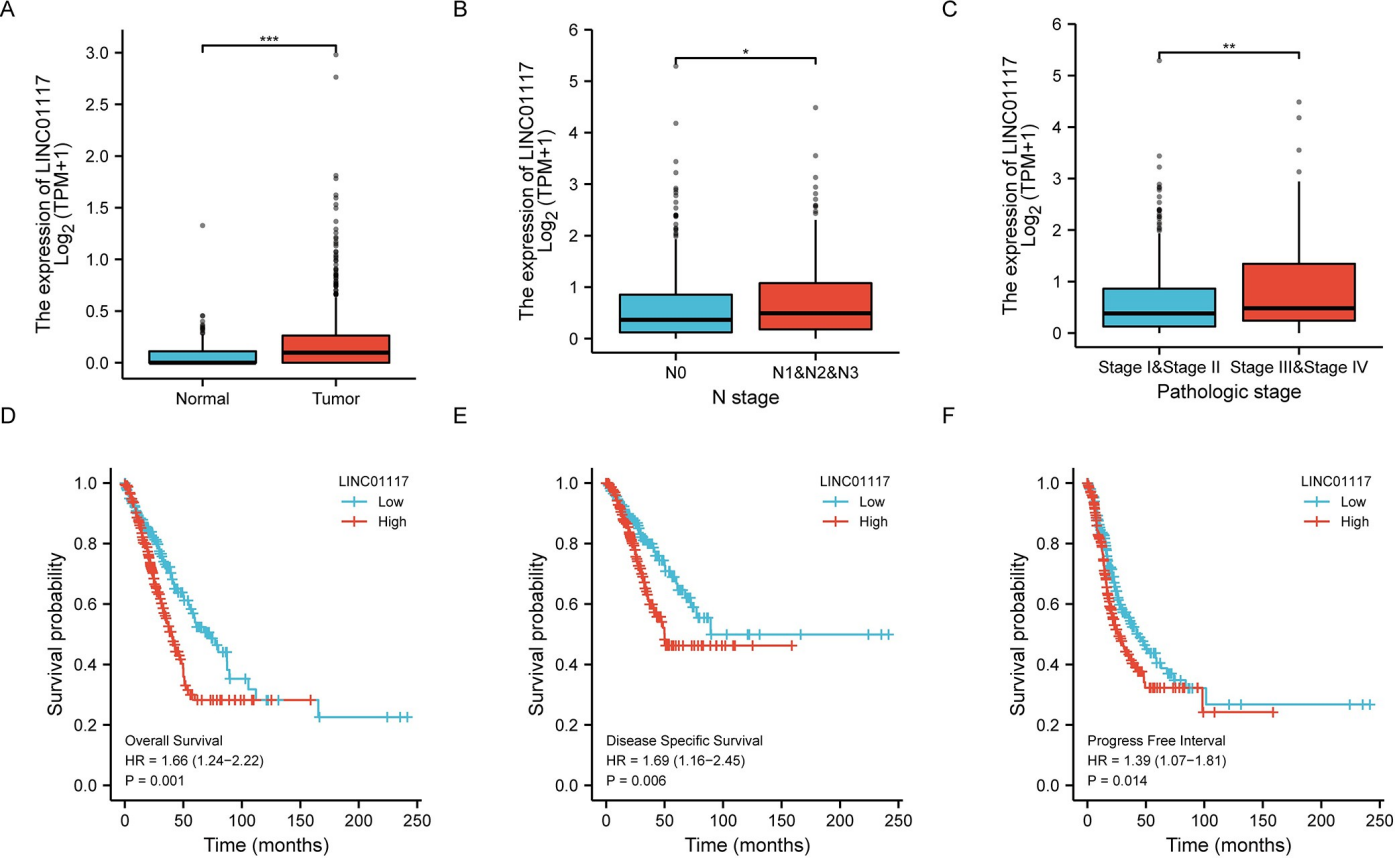
LINC01117 is upregulated in lung adenocarcinoma tissues and is associated with worse clinical features. (A) LINC01117 is upregulated in lung adenocarcinoma tissues. (B, C) Association of LINC01117 with clinical staging. (D-F) Association of LINC01117 with patient OS, DSS and PFI.

According to TCGA data, LINC01117 was significantly upregulated in LUAD tissues (Fig 1A). patients with elevated LINC01117 levels had poorer clinical characteristics, including clinical stage and N classification (Fig 1B and 1C), with higher stage associated with higher LINC01117 expression; patients with elevated LINC01117 levels had significantly shorter overall survival (OS), disease specific survival (DSS) and progression-free interval (PFI) were significantly shorter in patients with elevated LINC01117 levels (Fig 1D–1F, OS, HR = 1.66, p = 0.001; DSS, HR = 1.69, p = 0.006; PFI, HR = 1.39, p = 0.014). Univariate and multivariate analyses indicated that, independent of other clinical characteristics, LINC01117 was a valid prognostic biomarker Table 1.

**Table 1:** Univariate and multivariate analyses showed that LINC01117 was an independent prognostic marker for patients with lung cancer.

Characteristics	Total(N)	Univariate analysis		Multivariate analysis	
		Hazard ratio (95% CI)	P value	Hazard ratio (95% CI)	P value
T stage	**523**				
T1&T2	**457**	Reference			
T3&T4	**66**	2.317 (1.591–3.375)	**<0.001**	1.804 (1.134–2.869)	**0.013**
N stage	**510**				
N0	**343**	Reference			
N1&N2&N3	**167**	2.601 (1.944–3.480)	**<0.001**	2.244 (1.523–3.307)	**<0.001**
M stage	**377**				
M0	**352**	Reference			
M1	**25**	2.136 (1.248–3.653)	**0.006**	1.547 (0.802–2.987)	0.193
Pathologic stage	**518**				
Stage I&Stage II	**411**	Reference			
Stage III&Stage IV	**107**	2.664 (1.960–3.621)	**<0.001**	1.328 (0.815–2.164)	0.254
Gender	**526**				
Female	**280**	Reference			
Male	**246**	1.070 (0.803–1.426)	0.642		
Age	**516**				
< = 65	**255**	Reference			
>65	**261**	1.223 (0.916–1.635)	0.172		
LINC01117	**526**				
Low	**265**	Reference			
High	**261**	1.656 (1.236–2.220)	**<0.001**	1.828 (1.292–2.585)	**<0.001**

### LINC01117 is highly expressed in LUAD cells

**Fig 2:**
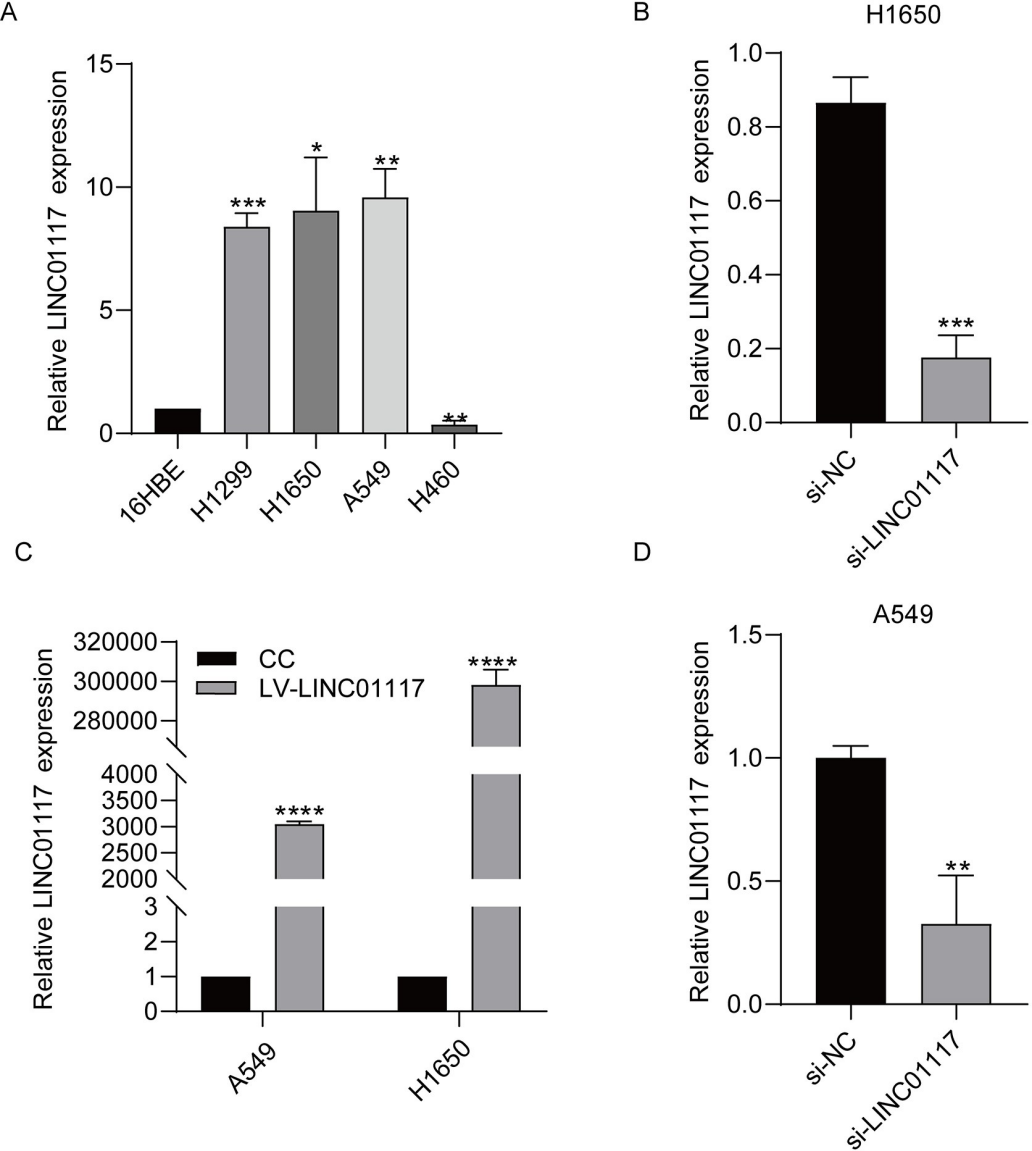
Expression of LINC01117 in lung adenocarcinoma cells. (A) Expression of LINC01117 in lung cancer cells. (B) Knockdown efficiency of LINC01117 in 1650 cells. (C) Expression of LINC01117 after overexpression. (D) Knockdown efficiency of LINC01117 in A549 cells.

We examined the role of LINC01117 in LUAD at the cellular level. We used RT-qPCR assays to validate LINC01117 expression levels in non-small cell carcinoma lung cancer and immortalized normal epithelial tissue cells, and showed that LINC01117 expression levels were significantly higher in LUAD cell lines H1650 and A549x than in normal human bronchial epithelial cells 16HBE, while in large cell carcinoma H1299 and H460 cell lines were inconsistent in expression (Fig 2A), so we selected LUAD cells H1650 and A549 cells for the follow-up study. The knockdown efficiency of siRNA and overexpression efficiency after lentiviral infection were then verified by RT-qPCR experiments, with knockdown efficiency reaching 70%-80% (Fig 2B and 2D) and overexpression ploidy reaching 3×103 and 3×105 (Fig 2C).

### LINC01117 affects the migration of LUAD cells

**Fig 3:**
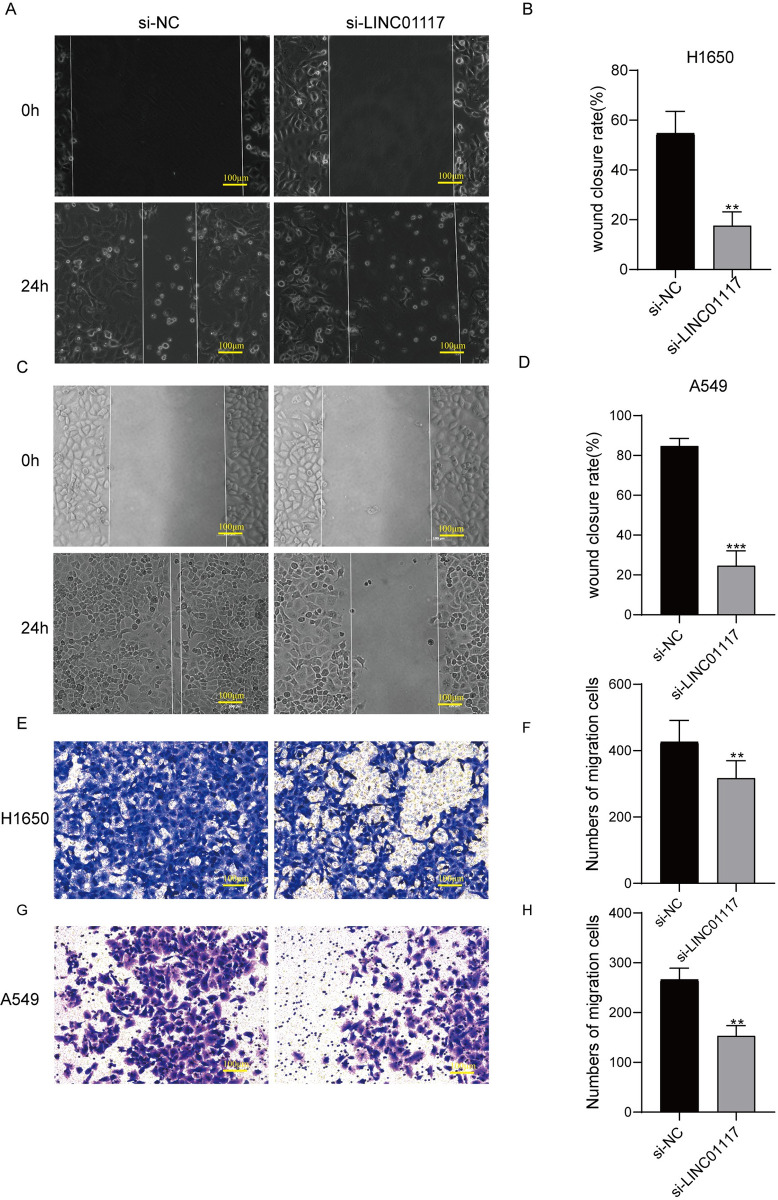
LINC01117 knockdown inhibits lung adenocarcinoma cell migration (A, B) Wound healing assay results for H1650 cells. (C, D) Wound healing assay results of A549 cells. (E, F) Transwell assay results of H1650 cells. (G, H) Transwell assay results of A549 cells.

**Fig 4:**
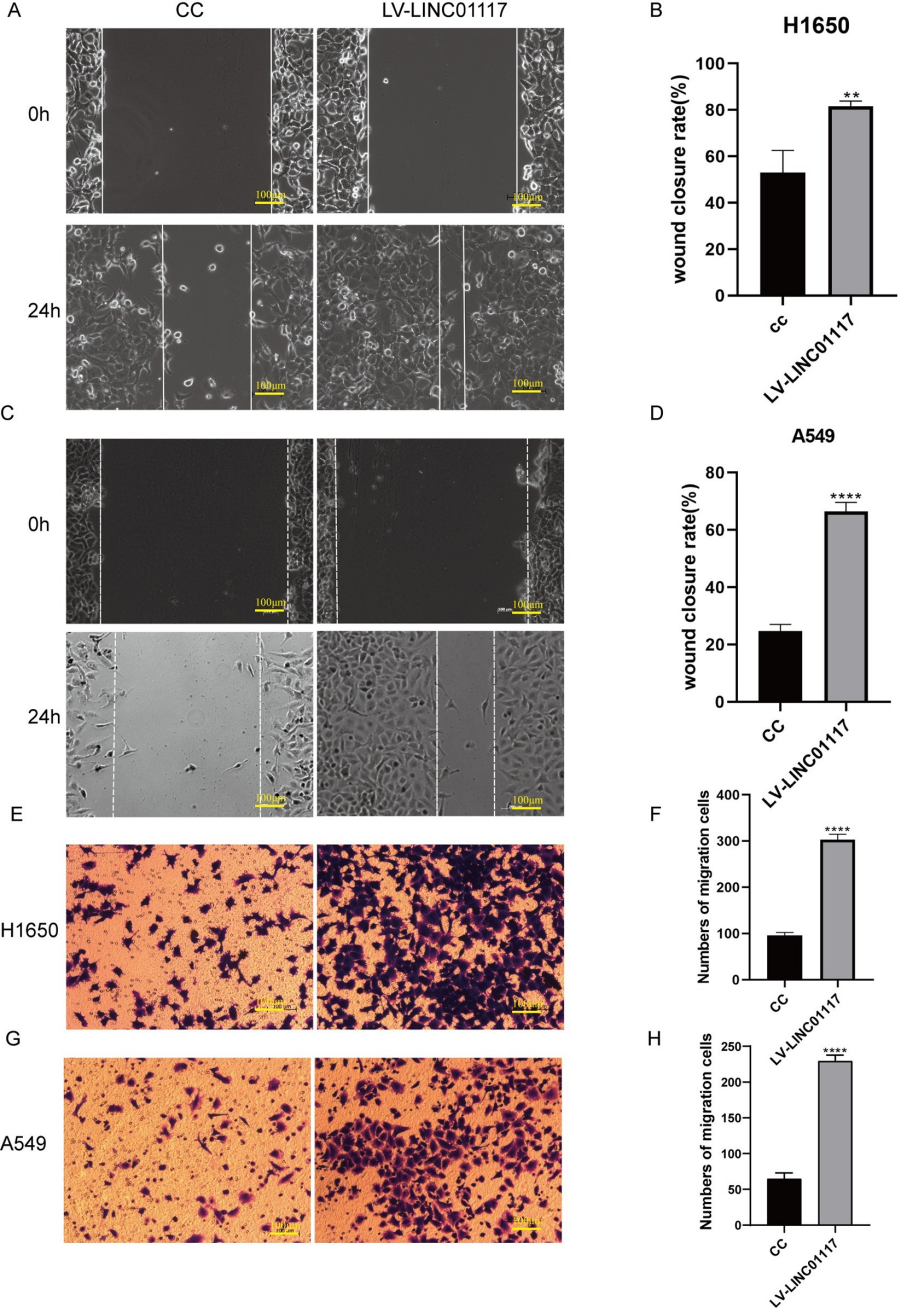
LINC01117 overexpression promotes migration of lung adenocarcinoma cells (A, B) Wound healing assay results for H1650 cells. (C, D) Wound healing assay results of A549 cells. (E, F) Results of Transwell assay on H1650 cells. (G, H) Results of Transwell assay on A549 cells.

Transwell migration assay and wound healing assay were used to examine the changes in cell migration ability after LINC01117 downregulation and overexpression. In the wound healing assay, the cell migration area of the LINC01117 knockdown group was significantly smaller than that of the negative control group (Fig 3A–3D), and the cell migration area of the LINC01117 gene overexpression group was significantly larger than that of the negative control group (Fig 4A–4D); in the Transwell assay, the number of cells migrating to the lower chamber of the LINC01117 knockdown group was significantly smaller than that of the negative In the Transwell assay, the number of cells migrating to the lower chamber of the LINC01117 knockdown group was significantly less than that of the negative control group (Fig 3E and 3F), and the number of cells migrating to the lower chamber of the LINC01117 knockdown group was significantly more than that of the negative control group (Fig 4E and 4F). The results showed that knocking down the expression level of LINC01117 inhibited the in vitro migration of H1650 and A549 cells; on the contrary, the in vitro migration ability of H1650 and A549 cells increased after overexpression of LINC01117.

### LINC01117 affects invasion of LUAD cells

**Fig 5:**
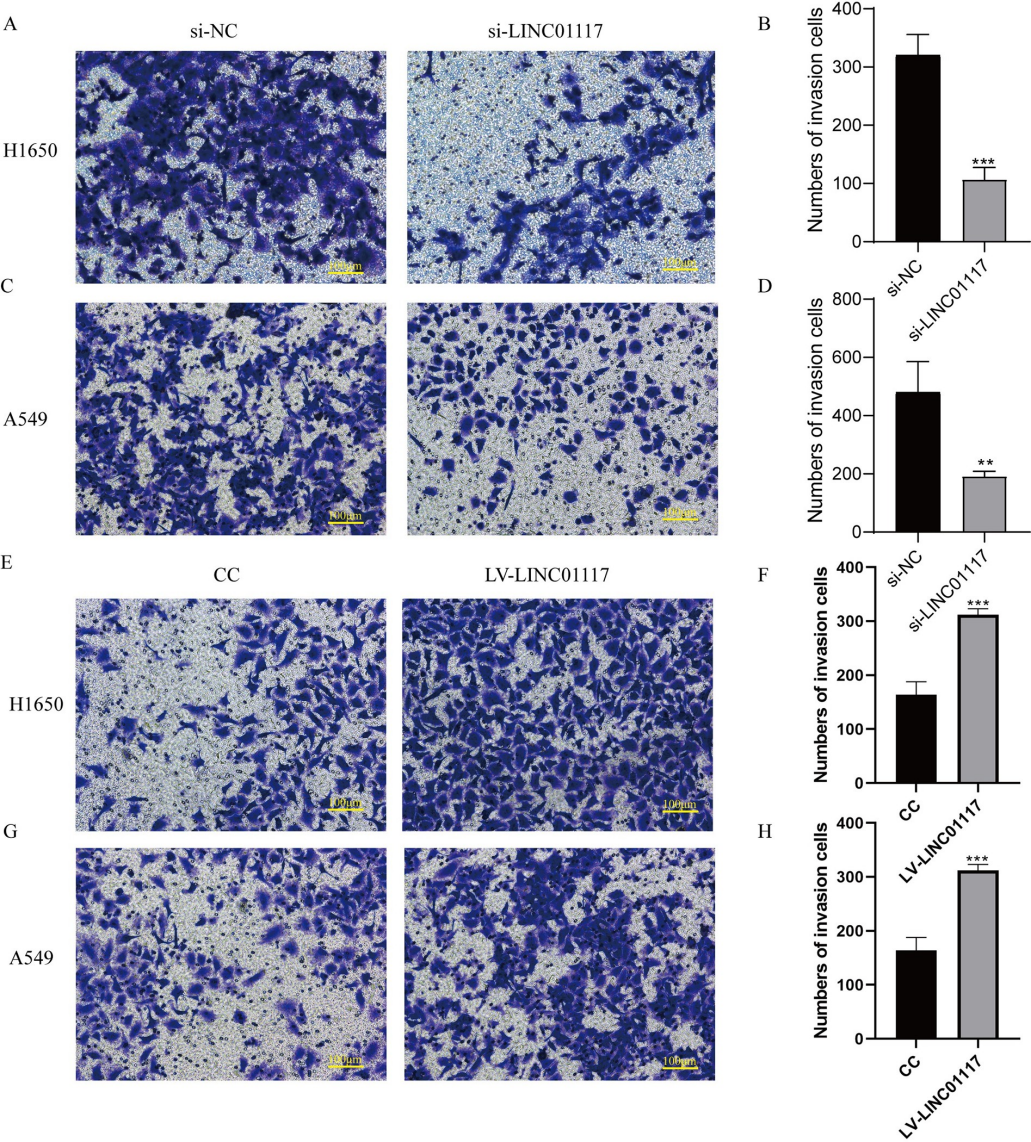
LINC01117 promotes lung adenocarcinoma cell invasion (A-D) Transwell invasion of H1650 and A549 cells after knockdown of LINC01117. (E-H) Transwell invasion results of H1650 and A549 cells after overexpression of LINC01117.

Transwell assays examined changes in cell invasion ability after LINC01117 knockdown and overexpression. The number of H1650 and A549 cells invading into the lower lumen was significantly less in the LINC01117 knockdown group than in the control group (Fig 5A–5D), while the number of H1650 and A549 cells invading into the lower lumen was significantly more in the LINC01117 overexpression group than in the control group (Fig 5E–5H). This indicates that knocking down the expression level of LINC01117 inhibited the invasion of H1650 and A549 cells in vitro; Conversely, the invasion ability of H1650 and A549 cells increased after overexpression of LINC01117.

### LINC01117 affects the EMT process

**Fig 6:**
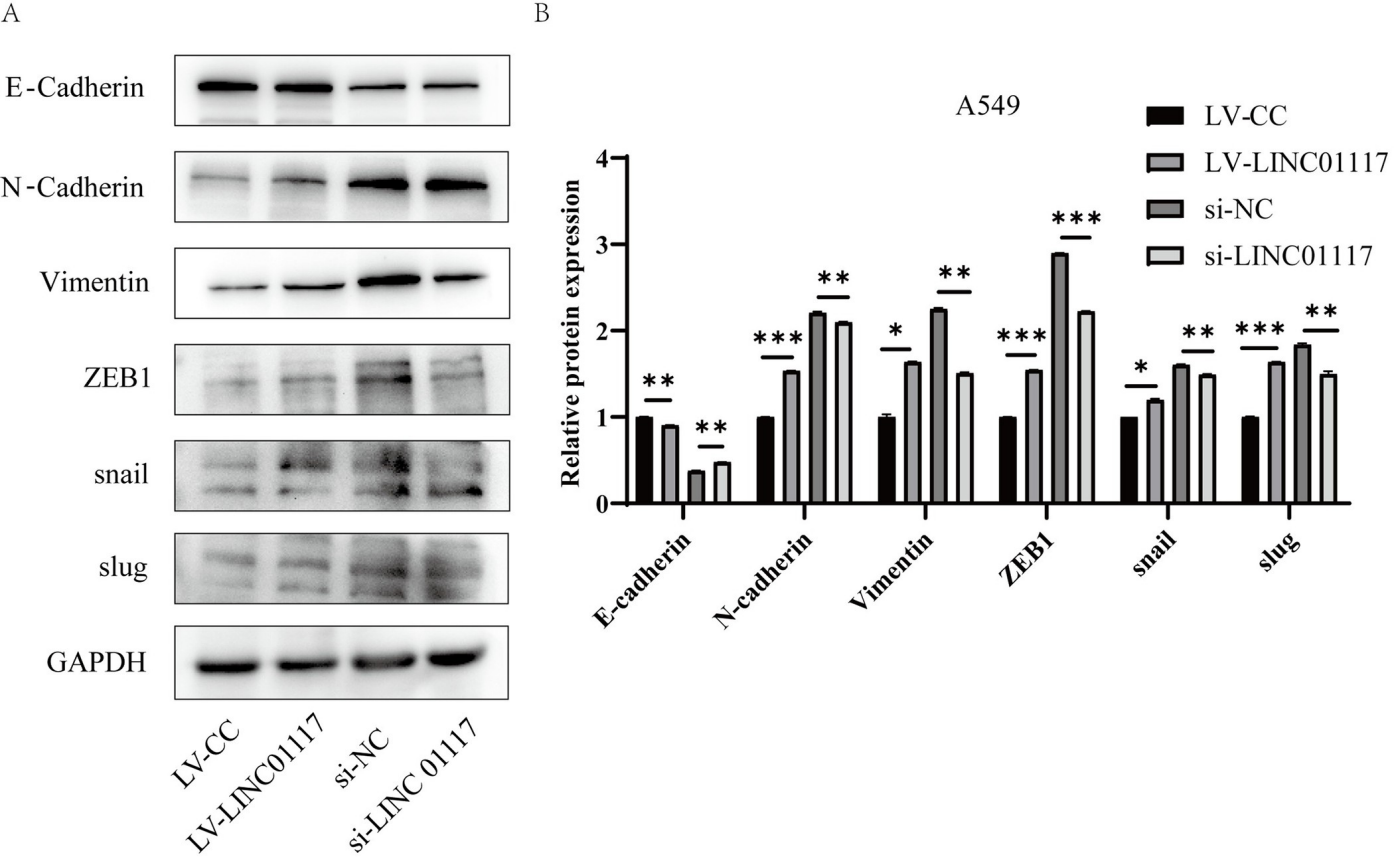
LINC01117 affects the EMT process (A, B) Western blot assays examined the expression of key proteins of the EMT process in control and LINC01117 overexpression groups of A549 cells.

In tumors metastasis, epithelial mesenchymal transition (EMT) is considered to be one of the important molecular mechanisms. To investigate in depth the molecular mechanisms by which LINC01117 promotes metastasis in LUAD cells, we next explored the role that LINC01117 plays in the EMT process in LUAD. We examined the expression of E-cadherin, N-cadherin, Vimentin, ZEB1, snail and slug, proteins related to the EMT pathway, in A549 cells overexpressing and knocking down LINC01117 by Western blot. The results showed that overexpression of LINC01117 significantly elevated the expression of N-cadherin, Vimentin, ZEB1, snail and slug, while the expression of E-cadherin was inhibited, and on the contrary, the opposite result was obtained in the knockdown group. (Fig 6A and 6B), These experimental results suggest a close correlation between changes in LINC01117 expression and the expression levels of EMT-related markers such as E-cadherin, N-cadherin and Vimentin, strongly suggesting that LINC01117 may regulate lung adenocarcinoma progression by affecting EMT process.

#### LINC01117 affects the nuclear and cytoplasmic distribution of YAP1 cells

**Fig 7:**
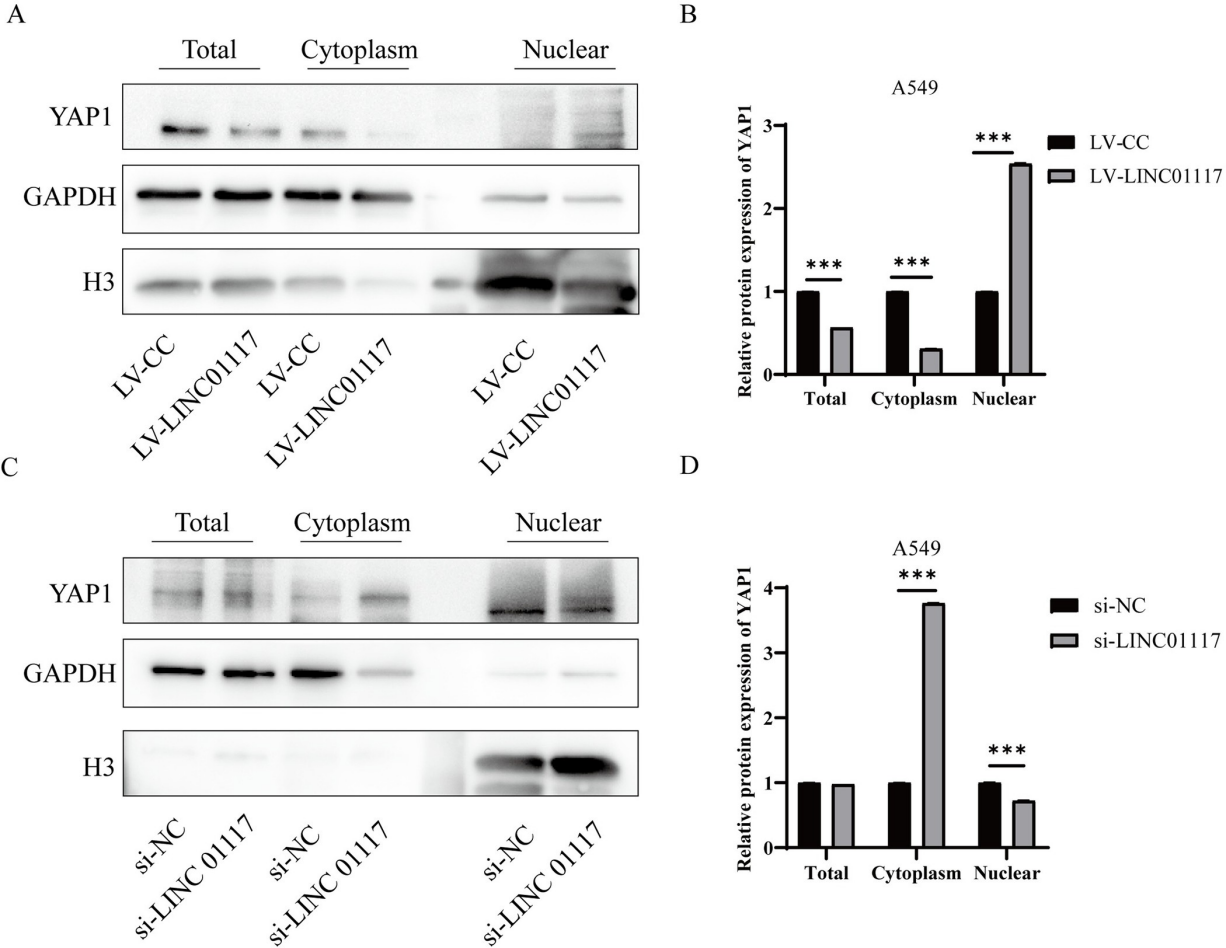
Effect of nuclear and cytoplasmic distribution of YAP1 in A549 cells overexpressing and knocking down LINC01117 (A, B) expression of YAP1 in the nucleus and cytoplasm in A549 cells overexpressing LINC01117; (C, D) expression of YAP1 in the nucleus and cytoplasm in A549 cells knocking down LINC01117.

The Hippo pathway has been widely demonstrated to play an important role in cancer, where YAP1, one of the key components of the Hippo pathway, interacts with EMT-related proteins to influence tumors cell migration and invasion. To investigate the role of LINC01117 in regulating the Hippo pathway in lung adenocarcinoma cells, we extracted total protein, cytoplasmic and nuclear proteins and assayed YAP1 levels in A549 cells with LINC01117 overexpression and knockdown. The results showed that although changes in LINC01117 expression did not significantly affect the overall intracellular levels of YAP1 protein, they significantly altered its distribution in the nucleus and cytoplasm, with knockdown of LINC01117 increasing the enrichment of YAP1 protein in the cytoplasm and decreasing its levels in the nucleus; whereas LINC01117 overexpression produced the opposite intracellular distribution results (Fig 7A and 7B). This suggests that LINC01117 may influence Hippo pathway activity by regulating the distribution of YAP1 in the nucleus and cytoplasm.

## Discussion

LUAD is a highly prevalent malignancy with a trend of increasing morbidity and mortality year by year. Although a combination of surgical treatment, radiotherapy, targeted therapy and immunotherapy has made considerable progress in the treatment of LUAD, metastasis remains the main cause of death in patients with LUAD [17]. Despite the extensive research in the direction of LUAD invasion and metastasis, few stable biomarkers have been identified and used to assess the risk of LUAD metastasis or to predict clinical outcomes. Therefore, the identification of validated key genes associated with LUAD invasion and metastasis is important for early diagnosis and improving the prognosis and clinical outcomes of LUAD patients. In recent years, an increasing number of aberrantly expressed genes have been identified to play a key role in the development and progression of various cancers, and LncRNAs can act as both oncogenes and tumors suppressor genes to regulate tumorigeneses and progression [17, 18]. Therefore, exploring the functions and mechanisms of action of differentially expressed LncRNAs in LUAD may lay the foundation for new diagnostic and therapeutic approaches for LUAD.

EMT is a mechanism that was first discovered in the 1980s [19]. EMT is the transition of cells from an epithelial to a mesenchymal state [20, 21]. This process modifies cell-expressed adhesion molecules, including E-cadherin, which is responsible for tight junctions, and the miRNA200 family, which helps maintain the epithelial phenotype. As cells acquire mesenchymal markers, such as N-cadherin, wave proteins and fibronectin, as well as the fibroblast proliferation transcription factors Snail, Slug and Twist, cells migrate towards a more mesenchymal phenotype [22]. EMT can be divided into three distinct types: type 1 EMT is an important physiological event during embryonic development and organogenesis; type 2 EMT is associated with adult tissue regeneration and occurs during wound healing, inducing cell migration, growth and organ fibrosis; and type 3 occurs during cancer progression [23, 24]. The miRNA-200 family and miR-205 together regulate the E-cadherin transcriptional repressors ZEB1 and SIP1, and decreased expression of these miRNAs upregulates ZEB1 and SIP1 [25], inhibits E-cadherin expression, and induces EMT [26]. The EMT process is regulated by multiple signaling pathways and transcriptional mechanisms, including LncRNAs [27]. Hu et al. found more than 99 LncRNAs involved in the EMT process [28]. Wang et al. found that MIR99AHG represses EMT in pulmonary fibrosis via the miR-136-5p/USP4/ACE2 axis [29]. Li et al. validated that LncRNA PCBP1-AS1 inhibited EMT progression suppressing LUAD metastasis [30]. In addition, a number of studies have found that LncRNAs can also regulate the EMT process in tumors through their role in related signaling pathways.

We found corresponding changes in the expression of EMT-related proteins in A549 cells overexpressing and knocking down LINC01117. Overexpression of LINC01117 promoted the EMT process; while knockdown of LINC01117 inhibited the EMT process, suggesting that LINC01117 in lung adenocarcinoma cells may promote cell migration and invasion through the EMT process. The Hippo pathway and the EMT process are inextricably linked [31, 32], and in particular, YAP1, a key effector of the Hippo pathway, can regulate EMT transcription factors through direct or indirect interactions to influence tumors cell invasion and metastasis [33]. In the case of Hippo pathway inhibition, YAP1 enters the nucleus and binds to transcription factors to promote transcriptional expression of downstream genes and influence tumors progression [34]. To investigate the effect of LINC01117 on the Hippo pathway, we extracted total, cytoplasmic and nuclear proteins from A549 cells with overexpression and knockdown of LINC01117, and examined YAP1 expression. The results suggest that LINC01117 may affect the migration and invasion of lung adenocarcinoma cells by influencing the expression of EMT transcription factors through the entry of YAP1 into the nucleus, providing a potential target for the treatment of LUAD.

## Supporting information

S1 Raw images (PDF)

## References

[1] Siegel RL, Miller KD, Fuchs HE, Jemal A. Cancer Statistics, 2021. CA: A Cancer Journal for Clinicians. 2021;71(1):7–33. doi: 10.3322/caac.21654 33433946

[2] Sung H, Ferlay J, Siegel RL, Laversanne M, Soerjomataram I, Jemal A, et al. Global Cancer Statistics 2020: GLOBOCAN Estimates of Incidence and Mortality Worldwide for 36 Cancers in 185 Countries. CA: A Cancer Journal for Clinicians. 2021;71(3):209–49. doi: 10.3322/caac.21660 33538338

[3] Schabath MB, Cote ML. Cancer Progress and Priorities: Lung Cancer. Cancer Epidemiology, Biomarkers & Prevention. 2019 2019-10-1;28(10):1563–79. doi: 10.1158/1055-9965.EPI-19-0221 31575553 PMC6777859

[4] Andrew G Nicholson MSTM. The 2021 WHO Classification of Lung Tumors: Impact of Advances Since 2015. J THORAC ONCOL. 2022;17(3):362–87. doi: 10.1016/j.jtho.2021.11.003 34808341

[5] Stinchcombe TE, Bogart J, Veeramachaneni NK, Kratzke R, Govindan R. Annual Review of Advances in Non-small Cell Lung Cancer Research: A Report for the Year 2010. J THORAC ONCOL. 2011 2011-1-1;6(8):1443–50. doi: 10.1097/JTO.0b013e3182246413 21709589

[6] Herbst RS, Morgensztern D, Boshoff C. The biology and management of non-small cell lung cancer. NATURE. 2018;553(7689):446–54. doi: 10.1038/nature25183 29364287

[7] Kim CFB, Jackson EL, Woolfenden AE, Lawrence S, Babar I, Vogel S, et al. Identification of Bronchioalveolar Stem Cells in Normal Lung and Lung Cancer. CELL. 2005;121(6):823–35. doi: 10.1016/j.cell.2005.03.032 15960971

[8] Fedorova AASA Topolnitskyc TSGE, Durovaf NASO Marina V. Zavyalovae FVMP. Prognosis of Different Types of Non-Small Cell Lung Cancer Progression: Current State and Perspectives. Cellular Physiology and Biochemistry. 2021 2021-3-10;55(S2):29–48. doi: 10.33594/000000340 33687819

[9] Park HJ, Lee SH, Chang YS. Recent advances in diagnostic technologies in lung cancer. The Korean Journal of Internal Medicine. 2020 2020-3-1;35(2):257–68. doi: 10.3904/kjim.2020.030 32131569 PMC7060993

[10] Cheng B, Xiong S, Li C, Liang H, Zhao Y, Li J, et al. An annual review of the remarkable advances in lung cancer clinical research in 2019. J THORAC DIS. 2020;12(3):1056–69. doi: 10.21037/jtd.2020.03.11 32274174 PMC7139050

[11] Ge X, Li G, Jiang L, Jia L, Zhang Z, Li X, et al. Long noncoding RNA CAR10 promotes lung adenocarcinoma metastasis via miR-203/30/SNAI axis. ONCOGENE. 2019;38(16):3061–76. doi: 10.1038/s41388-018-0645-x 30617305 PMC6484688

[12] Garbo S, Maione R, Tripodi M, Battistelli C. Next RNA Therapeutics: The Mine of Non-Coding. International Journal of Molecular Sciences. 2022 2022-7-5;23(13):7471. doi: 10.3390/ijms23137471 35806476 PMC9267739

[13] Derrien T, Johnson R, Bussotti G, Tanzer A, Djebali S, Tilgner H, et al. The GENCODE v7 catalog of human long noncoding RNAs: Analysis of their gene structure, evolution, and expression. GENOME RES. 2012;22(9):1775–89. doi: 10.1101/gr.132159.111 22955988 PMC3431493

[14] Flynn RA, Chang HY. Long Noncoding RNAs in Cell-Fate Programming and Reprogramming. CELL STEM CELL. 2014;14(6):752–61. doi: 10.1016/j.stem.2014.05.014 24905165 PMC4120821

[15] Morlando M, Fatica A. Alteration of Epigenetic Regulation by Long Noncoding RNAs in Cancer. International Journal of Molecular Sciences. 2018 2018-2-14;19(2):570. doi: 10.3390/ijms19020570 29443889 PMC5855792

[16] Wang H, Shu L, Niu N, Zhao C, Lu S, Li Y, et al. Novel lncRNAs with diagnostic or prognostic value screened out from breast cancer via bioinformatics analyses. PEERJ. 2022 2022-7-14;10:e13641. doi: 10.7717/peerj.13641 35855425 PMC9288825

[17] Xiaobin Guo ZCLZ. Long non-coding RNA-HAGLR suppressed tumor growth of lung adenocarcinoma through epigenetically silencing E2F1. EXP CELL RES. 2019;382(1). 10.1016/j.yexcr.2019.06.006 31194977

[18] Lizhong Zeng XLJY. Long non-coding RNA LINC01116 is overexpressed in lung adenocarcinoma and promotes tumor proliferation and metastasis. AM J TRANSL RES. 2020;12(8):4302–13. 32913506 PMC7476163

[19] G Greenburg EDH. Cytodifferentiation and tissue phenotype change during transformation of embryonic lens epithelium to mesenchyme-like cells in vitro. DEV BIOL. 1986;115(2):363–79. doi: 10.1016/0012-1606(86)90256-3 3519318

[20] Cui Y, Wang X, Zhang L, Liu W, Ning J, Gu R, et al. A novel epithelial-mesenchymal transition (EMT)-related gene signature of predictive value for the survival outcomes in lung adenocarcinoma. FRONT ONCOL. 2022 2022-9-15;12. doi: 10.3389/fonc.2022.974614 36185284 PMC9521574

[21] Bakir B, Chiarella AM, Pitarresi JR, Rustgi AK. EMT, MET, Plasticity, and Tumor Metastasis. TRENDS CELL BIOL. 2020;30(10):764–76. doi: 10.1016/j.tcb.2020.07.003 32800658 PMC7647095

[22] Zeisberg M, Neilson EG. Biomarkers for epithelial-mesenchymal transitions. J CLIN INVEST. 2009 2009-6-1;119(6):1429–37. doi: 10.1172/JCI36183 19487819 PMC2689132

[23] Raghu Kalluri RAW. The basics of epithelial-mesenchymal transition. J CLIN INVEST. 2009;119(6):1420–8. doi: 10.1172/JCI39104 19487818 PMC2689101

[24] Børretzen A, Gravdal K, Haukaas SA, Mannelqvist M, Beisland C, Akslen LA, et al. The epithelial–mesenchymal transition regulators Twist, Slug, and Snail are associated with aggressive tumour features and poor outcome in prostate cancer patients. The Journal of Pathology: Clinical Research. 2021;7(3):253–70. doi: 10.1002/cjp2.202 33605548 PMC8073012

[25] Kim NH, Song SH, Choi YH, Hwang KH, Yun JS, Song H, et al. Competing Endogenous RNA of Snail and Zeb1 UTR in Therapeutic Resistance of Colorectal Cancer. International Journal of Molecular Sciences. 2021 2021-9-3;22(17):9589. doi: 10.3390/ijms22179589 34502497 PMC8431469

[26] Senfter D, Madlener S, Krupitza G, Mader RM. The microRNA-200 family: still much to discover. Biomolecular Concepts. 2016;7(5–6):311–9. doi: 10.1515/bmc-2016-0020 27837593

[27] Liu T, Yang C, Wang W, Liu C. LncRNA SGMS1‐AS1 regulates lung adenocarcinoma cell proliferation, migration, invasion, and EMT progression viamiR‐106a‐5p/MYLI9 axis. THORAC CANCER. 2021;12(14):2104–12. doi: 10.1111/1759-7714.14043 34061466 PMC8287014

[28] Hu P, Yang J, Hou Y, Zhang H, Zeng Z, Zhao L, et al. LncRNA expression signatures of twist-induced epithelial-to-mesenchymal transition in MCF10A cells. CELL SIGNAL. 2014;26(1):83–93. doi: 10.1016/j.cellsig.2013.10.001 24113349

[29] Wang J, Xiang Y, Yang S, Zhang H, Li H, Zong Q, et al. MIR99AHG inhibits EMT in pulmonary fibrosis via the miR-136-5p/USP4/ACE2 axis. J TRANSL MED. 2022 2022-9-23;20(1). doi: 10.1186/s12967-022-03633-y 36138468 PMC9502606

[30] Li Z, Pan C, Wang Z, Deng X, Zhu Q, Wu W, et al. LncRNA PCBP1-AS1 correlated with the functional states of cancer cells and inhibited lung adenocarcinoma metastasis by suppressing the EMT progression. CARCINOGENESIS. 2021 2021-7-16;42(7):931–9. doi: 10.1093/carcin/bgab047 34107009

[31] Yu S, Zhang Y, Li Q, Zhang Z, Zhao G, Xu J. CLDN6 promotes tumor progression through the YAP1-snail1 axis in gastric cancer. CELL DEATH DIS. 2019 2019-1-1;10(12):949. doi: 10.1038/s41419-019-2168-y 31827075 PMC6906326

[32] Lehmann W, Mossmann D, Kleemann J, Mock K, Meisinger C, Brummer T, et al. ZEB1 turns into a transcriptional activator by interacting with YAP1 in aggressive cancer types. NAT COMMUN. 2016 2016-1-1;7(1):10498. doi: 10.1038/ncomms10498 26876920 PMC4756710

[33] Li H, Li Q, Jin M, Lu C, Mu Z, Xu W, et al. A review: hippo signaling pathway promotes tumor invasion and metastasis by regulating target gene expression. J CANCER RES CLIN. 2021 2021-1-1;147(6):1569–85. doi: 10.1007/s00432-021-03604-8 33864521 PMC11801896

[34] Yang S, Wang S, Chen L, Wang Z, Chen J, Ni Q, et al. Neutrophil Extracellular Traps Delay Diabetic Wound Healing by Inducing Endothelial-to-Mesenchymal Transition via the Hippo pathway. INT J BIOL SCI. 2023 2023-1-1;19(1):347–61. doi: 10.7150/ijbs.78046 36594092 PMC9760440

