# Supplementary material for: LINC01117 inhibits invasion and migration of lung adenocarcinoma through influencing EMT process
Source: PLoS One. 2023 Jun 29;18(6):e0287926. doi: 10.1371/journal.pone.0287926 (PMC10310029; doi:10.1371/journal.pone.0287926)
Supplement: S1 Raw images — (PDF) [file pone.0287926.s001.pdf]

Original images for blots:
A549 cells:
1

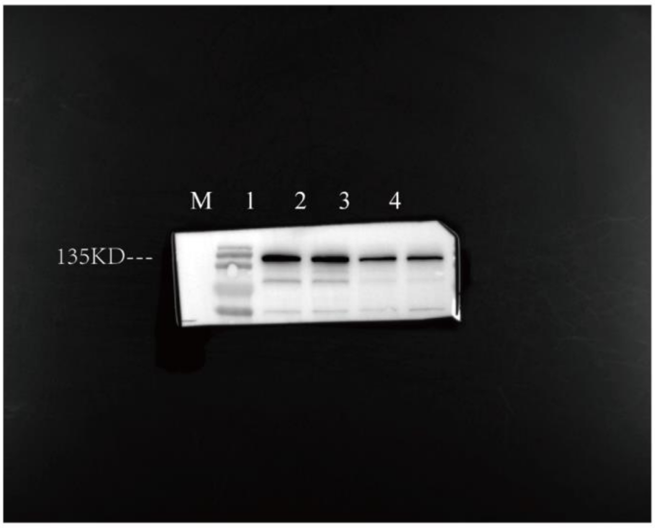

Protein：E-Cadherin
M：Marker （Epizyme , WJ 102）
1:LV-CC
2:LV-LINC01117
3:si-NC
4:si-LINC01117

Method used to capture the image:
Tanon 5200 Chemiluminescence Imager(Thermo Corporation,USA)
Figure panel: Fig 6A E-Cadherin

2

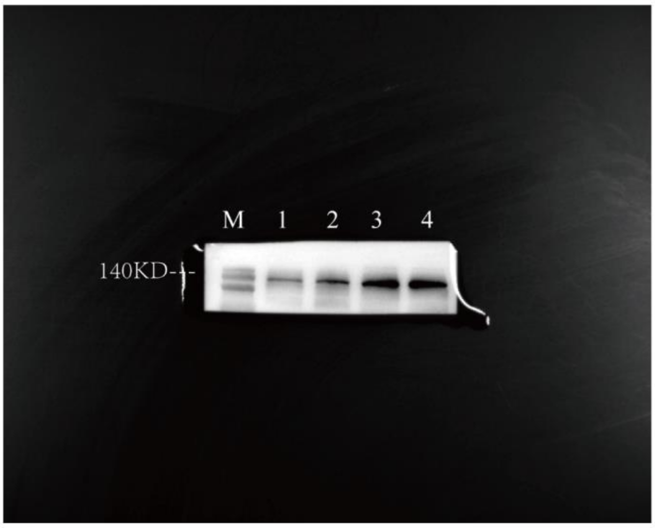

Protein：N-Cadherin
M：Marker （Epizyme , WJ 102）
1:LV-CC
2:LV-LINC01117
3:si-NC
4:si-LINC01117

Method used to capture the image:
Tanon 5200 Chemiluminescence Imager(Thermo Corporation,USA)
Figure panel: Fig 6A N-Cadherin

Original images for blots:
A549 cells:
3

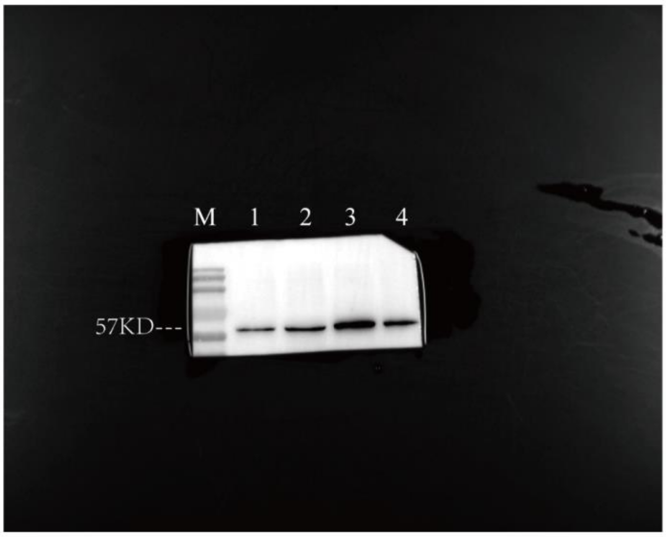

Protein： Vimentin
M： Marker （Epizyme , WJ 102）
1:LV-CC
2:LV-LINC01117
3:si-NC
4:si-LINC01117

Method used to capture the image:
Tanon 5200 Chemiluminescence Imager(Thermo Corporation,USA)
Figure panel: Fig 6A Vimentin

4

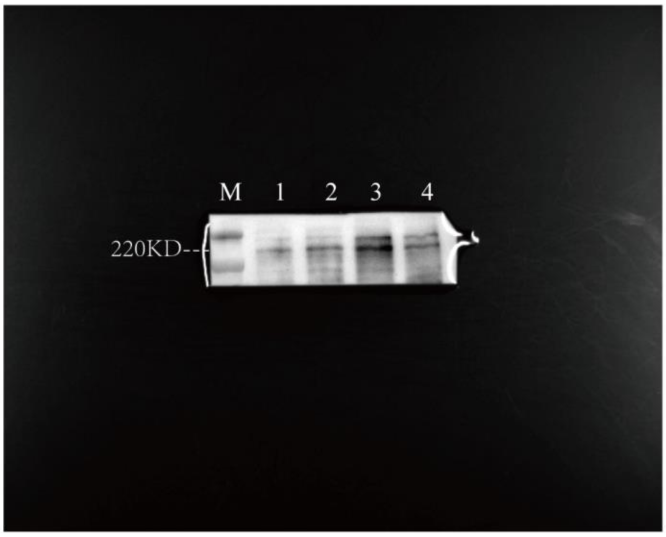

Protein： ZEB1
M： Marker （Epizyme , WJ 102）
1:LV-CC
2:LV-LINC01117
3:si-NC
4:si-LINC01117

Method used to capture the image:
Tanon 5200 Chemiluminescence Imager(Thermo Corporation,USA)
Figure panel: Fig 6A ZEB1

Original images for blots:
A549 cells:
5

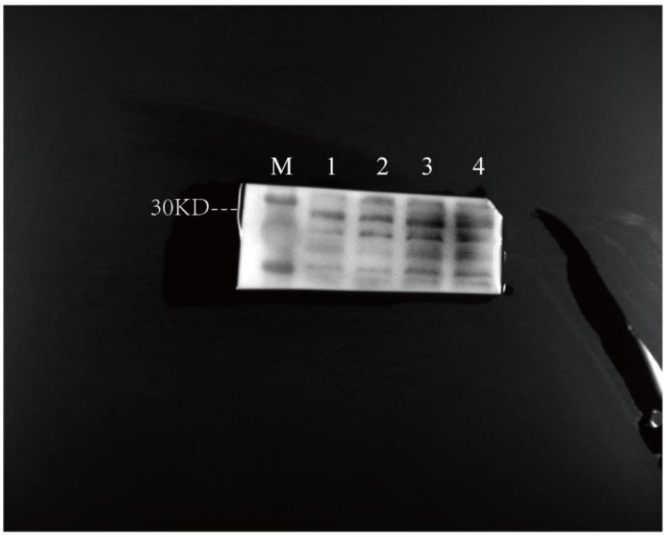

Protein： slug
M： Marker （Epizyme , WJ 102）
1:LV-CC
2:LV-LINC01117
3:si-NC
4:si-LINC01117

Method used to capture the image:
Tanon 5200 Chemiluminescence Imager(Thermo Corporation,USA)
Figure panel: Fig 6A slug

6

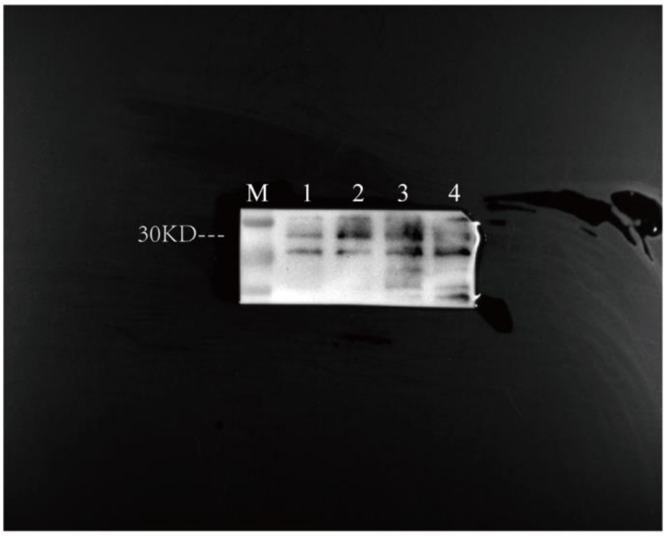

Protein： snail
M： Marker （Epizyme , WJ 102）
1:LV-CC
2:LV-LINC01117
3:si-NC
4:si-LINC01117

Method used to capture the image:
Tanon 5200 Chemiluminescence Imager(Thermo Corporation,USA)
Figure panel: Fig 6A snail

Original images for blots:
A549 cells:
7

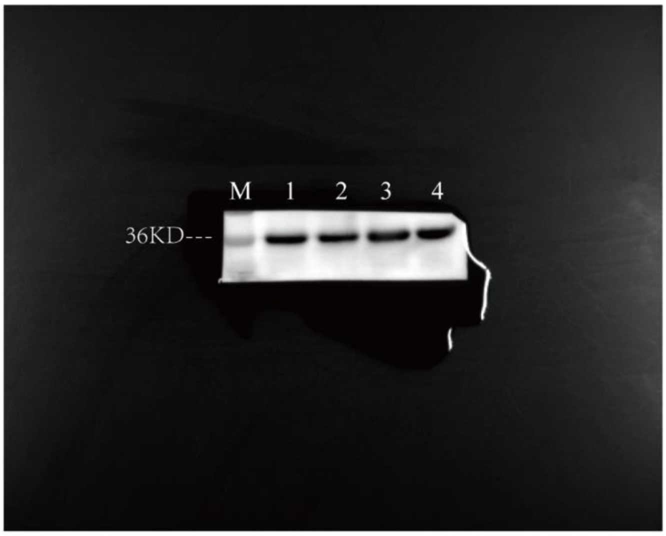

Protein： GAPDH
M： Marker （Epizyme , WJ 102）
1:LV-CC
2:LV-LINC01117
3:si-NC
4:si-LINC01117

Method used to capture the image:
Tanon 5200 Chemiluminescence Imager(Thermo Corporation,USA)
Figure panel: Fig 6A GAPDH

8

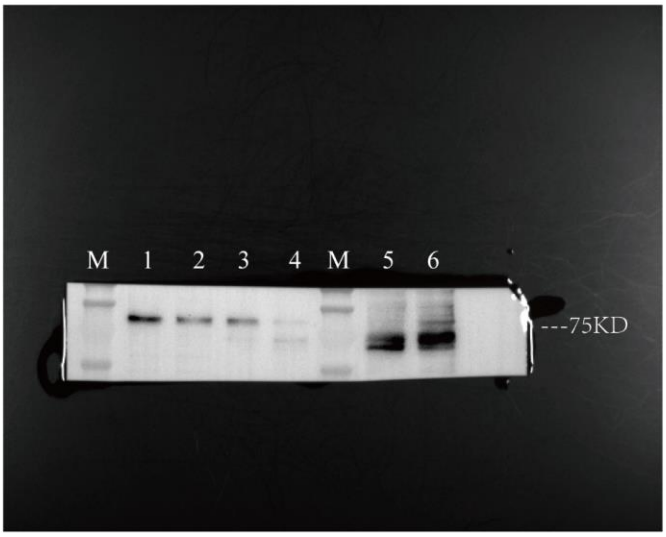

Protein： YAP1
M： Marker （Epizyme , WJ 102）
1:LV-CC Total
2:LV-LINC01117 Total
3:LV-CC Cytoplasm
4:LV-LINC01117 Cytoplasm
5:LV-CC Nuclear
6:LV-LINC01117 Nuclear

Method used to capture the image:
Tanon 5200 Chemiluminescence Imager(Thermo Corporation,USA)
Figure panel: Fig 7A YAP1

Original images for blots:
A549 cells:
9

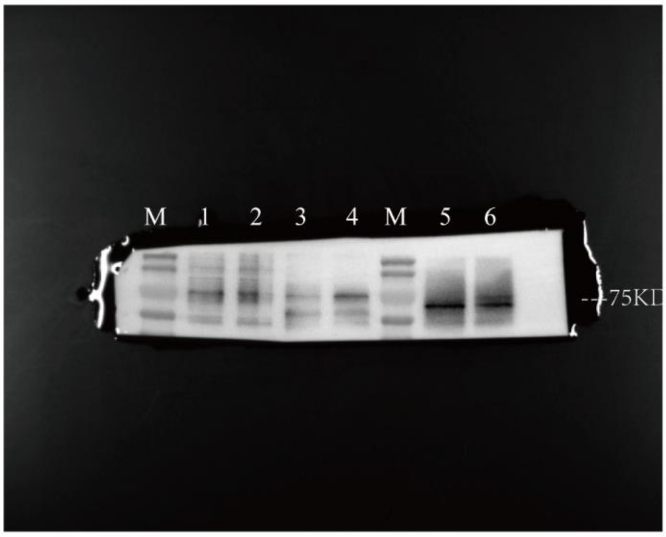

Protein: YAP1
M: Marker (Epizyme , WJ 102)
1:LV-CC Total
2:LV-LINC01117 Total
3:LV-CC Cytoplasm
4:LV-LINC01117 Cytoplasm
5:LV-CC Nuclear
6:LV-LINC01117 Nuclear

Method used to capture the image:
Tanon 5200 Chemiluminescence Imager(Thermo Corporation,USA)
Figure panel: Fig 7C YAP1

10

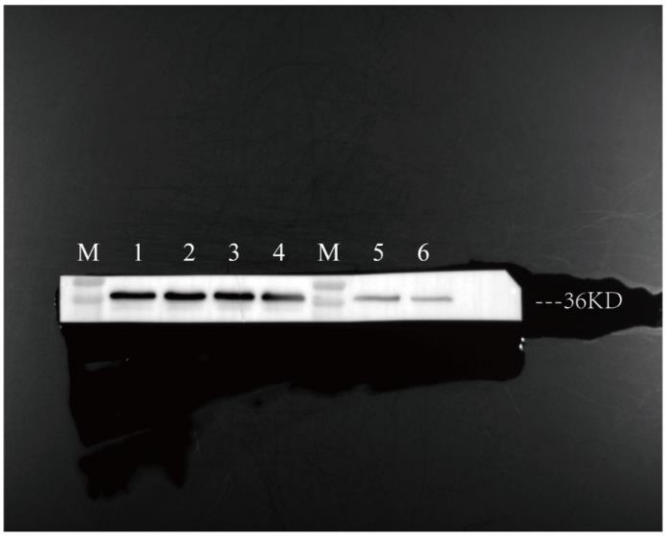

Protein: GAPDH
M: Marker (Epizyme , WJ 102)
1:LV-CC Total
2:LV-LINC01117 Total
3:LV-CC Cytoplasm
4:LV-LINC01117 Cytoplasm
5:LV-CC Nuclear
6:LV-LINC01117 Nuclear

Method used to capture the image:
Tanon 5200 Chemiluminescence Imager(Thermo Corporation,USA)
Figure panel: Fig 7A GAPDH

Original images for blots:
A549 cells:
11

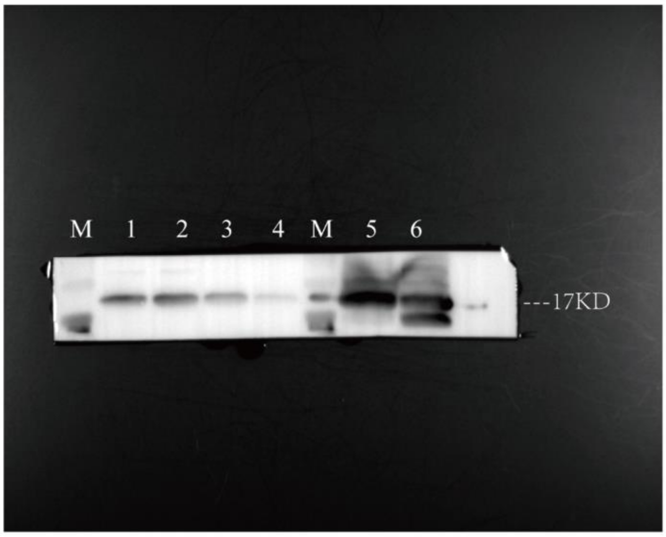

Protein：H3
M：Marker （Epizyme , WJ 102）
1:LV-CC Total
2:LV-LINC01117 Total
3:LV-CC Cytoplasm
4:LV-LINC01117 Cytoplasm
5:LV-CC Nuclear
6:LV-LINC01117 Nuclear

Method used to capture the image:
Tanon 5200 Chemiluminescence Imager(Thermo Corporation,USA)
Figure panel: Fig 7A H3

12

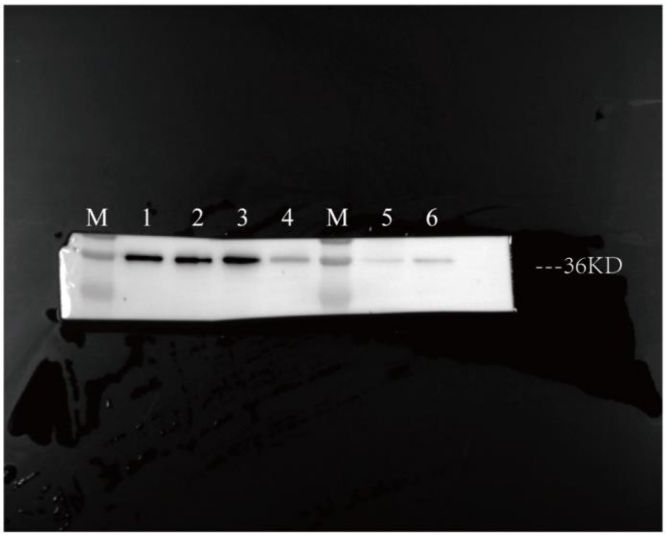

Protein：GAPDH
M：Marker （Epizyme , WJ 102）
1:LV-CC Total
2:LV-LINC01117 Total
3:LV-CC Cytoplasm
4:LV-LINC01117 Cytoplasm
5:LV-CC Nuclear
6:LV-LINC01117 Nuclear

Method used to capture the image:
Tanon 5200 Chemiluminescence Imager(Thermo Corporation,USA)
Figure panel: Fig 7C GAPDH

Original images for blots:
A549 cells:
13

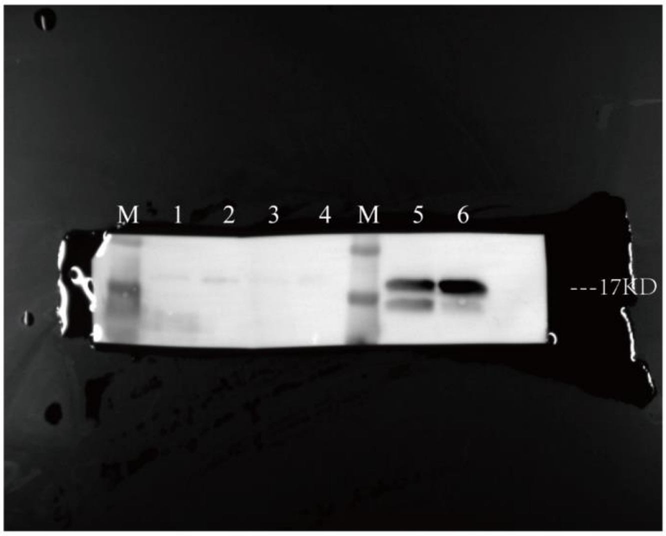

Protein：H3
M：Marker （Epizyme , WJ 102）
1:LV-CC Total
2:LV-LINC01117 Total
3:LV-CC Cytoplasm
4:LV-LINC01117 Cytoplasm
5:LV-CC Nuclear
6:LV-LINC01117 Nuclear

Method used to capture the image:
Tanon 5200 Chemiluminescence Imager(Thermo Corporation,USA)
Figure panel: Fig 7C H3
