# Supplementary material for: LINC01117 inhibits invasion and migration of lung adenocarcinoma through influencing EMT process
Source: PLoS One. 2023 Jun 29;18(6):e0287926. doi: 10.1371/journal.pone.0287926 (PMC10310029; doi:10.1371/journal.pone.0287926)
Supplement: Attachment — Submitted filename: Response to Reviewers.docx [file pone.0287926.s002.docx]

May 26, 2023

Dear Editor,

Many thanks for giving us the opportunity to revise the manuscript. We are appreciative of the detailed and constructive comments made by the reviewers. Based on these suggestions and comments, we have carefully revised the manuscript and highlighted the revised sections in the manuscript, which include the following: Firstly, we have revised the formatting of the text and references in accordance with the journal's requirements; subsequently we have revised and improved Figures 4-6 and added a new Figure 7 to explain the molecular mechanism of LINC01117 in lung adenocarcinoma. This was followed by adjustments to the English phrasing in the article. And in the discussion, we have added a discussion of the molecular mechanism of LINC01117 in lung adenocarcinoma. We hope that the revised version will adequately address the concerns of the reviewers.

The revised version was developed in consultation with all co-authors, each of whom gave their approval to the final form of the revised version. This manuscript has not been published or presented elsewhere, in part or in whole, nor has it been considered by another journal. There are no conflicts of interest to declare.

We thank you again for considering our revised manuscript. We look forward to hearing from you.

Kind Regards

Sincerely,

Liu Linjun

Beijing University of Technology

100 Pingle Yuan, Beijing 100124

P. R. China

Email: liulinjun816@126.com

**Response to Reviewer 1 Comments**

**Comment 1: Insufficient innovation in research, and no further research on the downstream regulatory mechanism of linc01117.**

Response 1: Thank you for your comment. In our newly submitted article, we complement the downstream regulatory mechanism of LINC01117 in lung adenocarcinoma, which may regulate Hippo pathway activity by altering the distribution of YAP1 in the nucleus and cytoplasm. (Please see lines 329 to 347)

**Comment 2: Is the position of the ruler in Figure 4c inconsistent? Was the image scaled?**

Response 2: Thank you very much for your careful identification of the problem with the scale in Figure 4. We have modified the image scales in Figure 4 and scaled the graphics consistently for better layout. We have also reconfirmed the scale of all the images. (Please see Fig 4)

**Comment 3: The protein band in Figure 6 does not match the quantification results. It is recommended to re-quantify using Image J.**

Response 3: Thank you for your comment. We have performed replicate experiments for Figure 6, in addition to validating the expression of the EMT-related proteins ZEB1, snail and slug proteins, and quantified them using Image J.(Please see Fig 6)

**Comment 4: The band of N-cadherin protein in Figure 6 seems to be a partial cut of a complete band. Please provide the image of original band.**

Response 4: Thank you for your comment. We have performed a repeat experiment for Figure 6 and uploaded the original uncropped and unadjusted images of all blot or gel result reports as required by the PLOS ONE journal. (Please see Fig 6 and S1_raw_images)

**Comment 5: The text and reference format does not meet the requirements of this journal.**

Response 4: Thank you for your comment. We deeply apologize for the issue of non-standard formatting in the manuscript. We have reread the manuscript upload requirements for the PLOS ONE journal and made modifications to the parts of the text and reference formats that do not comply with the requirements of this journal. (See highlighted sections in the text)

**Response to Reviewer 2 Comments**

The overall idea of the article is clear, the language is fluent, and the results are clear. Inhibition of LINC01117 expression can significantly inhibit the migration and invasion of lung adenocarcinoma cells. LINC01117 is associated with poor clinical features (stage and N classification) and poor prognosis, suggesting that LINC01117 may play a key role in the occurrence and development of lung adenocarcinoma. **The existing deficiencies are as follows:**

**Comment 1: General cell staining was performed for clone formation experiments, and entire Wells of 6-well plates were photographed. In this paper, the entire hole of the 6-well plate is not shown, and no scale is marked on the picture. The experimental images in FIG. 4 are of low quality and are not scaled.**

Response 1: Thank you for your comment. The clone formation experiment is not involved in this manuscript, and the staining images you see may be the experimental results of cell invasion and migration. We apologize for our carelessness regarding the lack of scale in the images. We have re standardized the scale and size of the images and adjusted their clarity. (Please see Fig 4)

**Comment 2: As for FIG5, it is too vague to see clearly when enlarged. The cell morphology is not clear and the background is a little messy. It is recommended to optimize the image processing method. In addition, there are too many cells in FIG5A and FIG5E, how to count them accurately? It is recommended to change to a graph with a moderate number of cells.**

Response 2: Thank you for your comment. Based on your suggestion, we have redone the experiment in Figure 5 to address issues such as unclear images and excessive cell density, and adjusted the clarity of the experimental results. As you mentioned, in the new experimental results graph, a moderate cell density is indeed more conducive to accurate counting. (Please see Fig 5)

**Comment 3: The number of bands in Western blot was too small, so it is suggested to increase the knockdown experiments before trend explanation.**

Response 3: Thank you for your comment. Based on your suggestion, we conducted knockdown experiments on LINC01117 and validated the levels of key proteins related to the EMT process through Western blot experiments. The results met our expectations and demonstrated that LINC01117 activated the EMT pathway. In addition, we validated the protein expression of other EMT related molecules ZEB1, Snail and Slug, and supplemented the above experimental results in the manuscript, increasing the credibility of our conclusion. (Please see Fig 6)

**Comment 4: The language of this article needs further modification.**

Response 4: Thank you for your comment. We deeply apologize for the language used in the manuscript. We spent a long time revising the manuscript and have highlighted it in yellow in the text. We have now conducted research on language and readability, and we really hope to have a substantial improvement in the language proficiency of the article.
